# Genome Sequence of a Wa-Like G3P[8] Rotavirus from a 12-Month-Old Child with Diarrhea in Manipur, India

**DOI:** 10.1128/mra.01254-21

**Published:** 2022-07-25

**Authors:** Yengkhom Damayanti Devi, Upalabdha Dey, Aditya Kumar, Chongtham Shyamsunder Singh, Nima D. Namsa

**Affiliations:** a Department of Molecular Biology and Biotechnology, Tezpur University, Napaam, Assam, India; b Department of Paediatrics, Regional Institute of Medical Sciences, Imphal, India; c Centre for Multi-disciplinary Research, Tezpur University, Napaam, Assam, India; KU Leuven

## Abstract

Rotavirus A (RVA) was detected in the stool of a 12-month-old child with diarrhea, mild fever, and vomiting. A viral metagenomic approach identified a Wa-like genotype G3P[8] strain named RVA/Human-wt/IND/RM25112/2016.

## ANNOUNCEMENT

Rotavirus A (RVA) is the major etiologic agent of acute gastroenteritis in young children worldwide. Six structural proteins (VP1 to VP4, VP6, and VP7) and six nonstructural proteins (NSPs) (NSP1 to NSP6) are encoded by the 11-double-stranded RNA (dsRNA) genome (1). The VP7-VP4-VP6-VP1-VP2-VP3-NSP1-NSP2-NSP3-NSP4-NSP5/NSP6 genes of an RVA strain are denoted by the descriptor Gx-P[x]-Ix-Rx-Cx-Mx-Ax-Nx-Tx-Ex-Hx (x represents the genotype number) in the classification system (2). The Wa, DS-1, and AU-1 genotype constellations are described as G1-P[8]-I1-R1-C1-M1-A1-N1-T1-E1-H1, G2-P[4]-I2-R2-C2-M2-A2-N2-T2-E2-H2, and G3-P[9]-I3-R3-C3-M3-A3-N3-T3-E3-H3, respectively (3–6).

The presence of RVA antigen in stool was confirmed using the Premier Rotaclone enzyme-linked immunosorbent assay (ELISA) kit (Meridian Bioscience Inc., USA) and a rapid immunochromatographic test, the Rota+Adeno+Astro+Noro enzyme immunoassay (EIA) combo card kit (CerTest Biotec S.L., Spain). The sample was homogenized by vortex-mixing at room temperature for 5 min, centrifuged at 14,500 rpm for 12 min, and filtered through a 0.22-μm filter. Total RNA was extracted using the TRIzol method (catalog number 15596018; Invitrogen, Carlsbad, CA, USA). cDNA was prepared using SuperScript III reverse transcriptase and random hexamers (Invitrogen). The dsRNA was denatured at 95°C for 5 min. The reverse transcription reaction was carried out with thermal cycling steps at 25°C for 10 min and 55°C for 60 min. VP7 and VP4 nested multiplex PCR and sequencing confirmed the isolate as the G3P[8] genotype (7).

The TruSeq stranded total RNA library preparation kit (catalog number 20020594; Illumina) was utilized to prepare the library for whole-genome sequencing, and final libraries were quantified using a Qubit v.4.0 fluorometer and a Qubit DNA high-sensitivity (HS) assay kit (catalog number Q32851; Thermo Fisher Scientific). Paired-end (2 × 150-bp) sequencing was performed using an Illumina NovaSeq 6000 system. A total of 19.6 million raw reads were generated; after trimming of the adapters and barcodes, 17.4 million retained reads were utilized for the downstream analysis. The quality control of the raw data was carried out using FastQC v.0.11.9. The processed reads were submitted to Genome Detective (8) and CCMetagen (9) servers to determine the taxonomic assignment of the sequences. The cleaned reads were aligned with rotavirus reference genome sequences obtained from NCBI (GenBank accession numbers GCA_003156295.1, GCA_003259085.1, GCF_000864225.1, GCF_000864245.1, GCF_000880735.1, GCF_000890155.1, GCF_000907835.1, GCF_000910335.1, GCF_000973395.3, GCF_001343825.1, GCF_004117615.1, and GCF_013086085.1), and the alignment statistics were measured using Bowtie2 v.2.4.2 (10). The rotavirus-specific reads were extracted using CCMetagen and *de novo* assembled with MEGAHIT v.1.2.9 (11). All software was used with default settings.

The CCMetagen protocol yielded 8.7 million reads after quality trimming. Eleven nearly complete genome segments of an RVA strain were recovered based on the length of each best hit, as shown in Table 1. This strain was typed as G3P[8] with a human Wa-like genotype constellation (I1-R1-C1-M1-A1-N1-T1-E1-H1) and a common ancestor with porcine RVA strains (5) using the ViPR (12) and RotaC (13) tools. All 11 segments showed a range of 96.93% to 99.49% similarity with known contemporary RVA strains (Table 1). This genome will serve as a reference for the region and form part of the baseline data that might be needed to address the impact of rotavirus vaccination in India (14).

**TABLE 1:** Assembly and genotyping details for the RVA genome described in this study

Segment no. (protein product)	Segment length (bp)	Mean coverage (×)	No. of reads	GC content (%)	ORF length (aa) (protein product)^*a*^	Genotype	GenBank accession no. for closest strain	GenBank accession no.	Similarity to closest strain (%)
1 (VP1)	3,299	871	6,270	31	955 (VP1)	R1	GU199492.1	OL906393	98.34
2 (VP2)	2,915	1,179	7,358	31	894 (VP2)	C1	DQ146661.1	OL906392	98.35
3 (VP3)	2,737	1,068	6,369	29	835 (VP3)	M1	DQ146651.1	OL906391	98.05
4 (VP4)	2,443	1,103	6,666	31	775 (VP4)	P[8]	DQ146652.1	OL906390	97.12
5 (NSP1)	1,673	927	3,533	29	497 (NSP1)	A1	EF560708.1	OL906389	98.08
6 (VP6)	1,438	1,799	6,405	36	397 (VP6)	I1	DQ146642.1	OL906388	98.83
7 (NSP3)	1,205	1,055	2,223	32	310 (NSP3)	T1	DQ146646.1	OL906387	97.32
8 (NSP2)	1,201	650	1,763	33	317 (NSP2)	N1	DQ146656.1	OL906386	98.32
9 (VP7)	1,189	971	2,247	34	326 (VP7)	G3	EF672602.1	OL906385	96.93
10 (NSP4)	922	951	2,017	38	175 (NSP4)	E1	DQ146658.1	OL906384	98.86
11 (NSP5/NSP6)	742	838	1,375	39	197 (NSP5)/90 (NSP6)	H1	DQ146681.1	OL906383	99.49

a ORF, open reading frame; aa, amino acids.

This study was approved by the institutional ethical review board of Tezpur University [approval number DoRD/TUEC/10-14/2017/4(b)], and written informed consent was obtained from the parents/guardians of study participants and control subjects.

### Data availability.

The assembled genomes have been deposited in GenBank under the accession numbers OL906383 to OL906393. The raw reads have been deposited under SRA accession number SRX14381993.
